# Muscle relaxation enhances motor imagery capacity in people with anxiety: A randomized clinical trial

**DOI:** 10.1371/journal.pone.0316723

**Published:** 2025-01-10

**Authors:** Sara Trapero-Asenjo, Sara Fernández-Guinea, Aymeric Guillot, Daniel Pecos-Martin, Susana Nunez-Nagy

**Affiliations:** 1 Department of Nursing and Physiotherapy, Faculty of Medicine and Health Sciences, University of Alcalá, Alcalá de Henares, Spain; 2 Humanization in the Intervention of Physiotherapy for the Integral Attention to the People Group (HIPATIA), University of Alcalá, Alcalá de Henares, Spain; 3 Health Technology Integration Research Group (GITES), Castilla-La Mancha Institute of Health Research, Toledo, Spain; 4 Department of Experimental Psychology, Cognitive Processes and Speech Therapy, Complutense University, Madrid, Spain; 5 Clinical Neuroscience Group, Complutense University, Madrid, Spain; 6 Laboratoire Interuniversitaire de Biologie de la Motricité, UCBL-Lyon 1, UR 7424, Universite Lyon 1, Villeurbanne, France; 7 Physiotherapy in the Approach to Pain, Telephysiotherapy and Augmented Therapeutic Reality Group, University of Alcalá, Alcalá de Henares, Spain; University Hospital Marques de Valdecilla, SPAIN

## Abstract

**Background:**

Motor imagery is the mental representation of a movement without physical execution. When motor imagery is performed to enhance motor learning and performance, participants must reach a temporal congruence between the imagined and actual movement execution. Identifying factors that can influence this capacity could enhance the effectiveness of motor imagery programs. Anxiety frequently occurs in sports and rehabilitation where motor imagery is a relevant technique. It is associated with increased muscle tension and impairs the memory processes involved in motor imagery. This study aimed to determine whether muscle relaxation before motor imagery practice can influence motor imagery capacity and temporal congruence in anxious individuals, during internal and external visual imagery, and kinesthetic imagery.

**Methods:**

A randomized clinical trial was conducted in 55 young adults (20.3±2.8 years; 40 females; 15 males) with anxiety (percentile ≥75% on the State-Trait Anxiety Inventory). 26 participants were assigned to the relaxation group and 29 to the control group through stratified randomization. Motor imagery capacity and temporal congruence were assessed using the Movement Imagery Questionnaire-3 at 2 points (t1 and t2). Between t1 and t2, participants in the relaxation group underwent abbreviated progressive relaxation training. Electrodermal activity and heart rate variability were recorded to evaluate the relaxation effect.

**Results:**

Data revealed a significant improvement in motor imagery capacity in the relaxation group, while the temporal congruence was not impaired in both groups.

**Conclusion:**

Pre-motor imagery muscle relaxation might improve motor imagery capacity in anxious individuals. This finding may contribute to better tailor motor imagery programs and to adjust motor imagery guidelines and recommendations for people with anxiety.

This study has been registered in ClinicalTrials.gov (NCT04973956).

## Introduction

Motor imagery (MI) is an increasingly used technique in sports and rehabilitation due to its effectiveness in enhancing motor learning and performance and promoting motor recovery [1, 2]. Consequently, there is huge interest in generating guidelines for effective MI interventions in both clinical and research settings [3]. MI involves the mental representation of an action or movement without actual body movement [1, 2]. It is a complex cognitive process that activates internal movement representations involving various memory mechanisms, including working memory [1]. There is also a functional correspondence between MI and actual movement execution as both have been extensively shown to activate overlapping brain regions [4, 5].

A key issue during MI practice is the quality or accuracy of the mental images generated. Various complementary approaches have been used to fully evaluate individual MI capacity [6–8]. On the one hand, the capacity to generate a clear and vivid mental image of the movement can be assessed through self-reported questionnaires, while the capacity to maintain temporal congruence (TC) between imagination and movement execution should also be examined [6]. Additionally, it is important to evaluate different capacities of internal visual imagery (IVI), external visual imagery (EVI), and kinesthetic imagery (KI). Although these imagery modalities are related constructs, neuroimaging data provided evidence that each is mediated by distinct neural networks [9, 10]. Engaging in MI training contribute to substantially develop these imagery modalities and further improve both imagery vividness and the ability to reach the temporal congruence [11]. As a matter of fact, MI capacity can improve [1], and when it does, motor performance following MI practice also seems to improve [12]. Thus, understanding factors that can maximize individual MI capacity might contribute to improve the effectiveness of MI programs.

Anxiety frequently appears in sports and rehabilitation settings, both during competitions [13] and recovery periods from injuries of different etiologies [14–16]. Anxiety is an emotional state characterized by apprehension and somatic symptoms of tension [17]. It is consistently associated with elevated muscle tension [18] and poorer working memory performance [19–21]. While anxiety can affect motor performance [22], previous data reported that anxiety might also impair the MI experience [1, 23]. Progressive relaxation training [24] and its abbreviated method, abbreviated progressive relaxation training (APRT) [25], were found to effectively reduce anxiety by decreasing muscle tension [18, 25, 26]. APRT promotes both physiological and psychological relaxation [27]. Additionally, this technique has shown improvements in working memory capacity [28]. One should thus argue that performing relaxation training is a relevant mean to manage anxiety before competitive events, and further preserve the ability to use MI efficiently.

The effects of pre-MI relaxation on movement imagination capacity have been scarcely explored. MI has first extensively been performed in a relaxed state, to remove external distractions, reduce somatic tensions, as well as to improve concentration and introspection [29–32]. Combining MI and relaxation techniques further contributed to increasing self-confidence and managing anxiety [33, 34], and to improve subsequent motor performance [35]. Morris et al. [36, p.188] even concluded that ‘‘*until researchers achieve greater clarification between relaxation and imagery*, *the consensus opinion would support including relaxation in imagery training programs*”. Some authors, however, later challenged the idea that relaxation is a systematic prerequisite for MI and argued that relaxation may even prevent from imagery-related benefits when the aim is to improve motor learning and performance [37]. Louis et al. [38] specifically explored whether arousal or relaxation before MI affected imagery vividness and execution and imagination times. Young athletes performed movements typical of their sport, all with similar difficulty. They then randomly performed independent MI sessions. At the start of each session, they were subjected to a 10-minute arousal situation (through intense physical practice), relaxation (by listening to a relaxation exercise), or a baseline condition (maintaining a conversation with the experimenter). They were then asked to imagine the same executed movements, combining IVI and KI, and execution and imagination times and imagery vividness were evaluated. Relaxation was found to substantially negatively affect TC between imagination and movement execution, while there were no differences in MI vividness [38]. Based on previous data demonstrating that it is imperative to control the MI timing to avoid detrimental effects on subsequent motor performance [39], these results support that pre-MI relaxation might thus be harmful. Kurtz [40] later explored whether mindfulness meditation affected MI vividness in young people. Participants randomly performed 2 different sessions on separate days. They first physically performed a precise target-throwing task. Then, in one session, they were subjected to 5 minutes of mindfulness before MI, and in another session, they only performed MI for the task. No differences in imagery vividness were observed whether or not mindfulness preceded MI [40]. This lack of effect of meditation on MI vividness was even confirmed when considering the level of anxiety of the participants as a covariable, as measured with the State-Trait Anxiety Inventory (STAI) [40]. The findings by Louis et al. [38] and Kurtz et al. [40] thus support the postulate that relaxation may be a starting point for the generation of vivid mental images. On the other hand, other authors argue that the state of relaxation should not be maintained during the entire MI session [31].

To our knowledge, no studies have yet explored whether pre-MI muscle relaxation through APRT might selectively enhance MI capacity in anxious individuals. However, muscle relaxation through APRT reduces anxiety and anxiety might impair MI. This study thus aims to answer the question: could pre-MI muscle relaxation influence MI capacity and TC during IVI, EVI, and KI, in anxious individuals? Anxious participants who undergo pre-MI muscle relaxation through APRT are expected to have greater MI capacity and TC between movement execution and imagination than those who did not undergo muscle relaxation.

## Materials and methods

### Study design

A double-blind randomized clinical trial was conducted. The participant did not know what the other participants are doing, and a researcher blind to the intervention analyzed the data to obtain the results. Randomization was performed through stratified allocation by sex to ensure homogeneous gender proportions in all groups. One researcher (SNN) performed a simple randomization [1:1] within each stratum to the intervention groups (relaxation or control) using the Epidat 4.2 program and placed the randomization result into sealed envelopes. Another researcher (STA) was responsible for enrolling participants, opening the envelopes at the time of the study, and conducting the experiment.

The study was approved by the Ethics Committee for Research and Animal Experimentation of the University of Alcalá (CEID2022/2/036) and registered in ClinicalTrials.gov (NCT04973956).

### Participants

**Fig 1:**
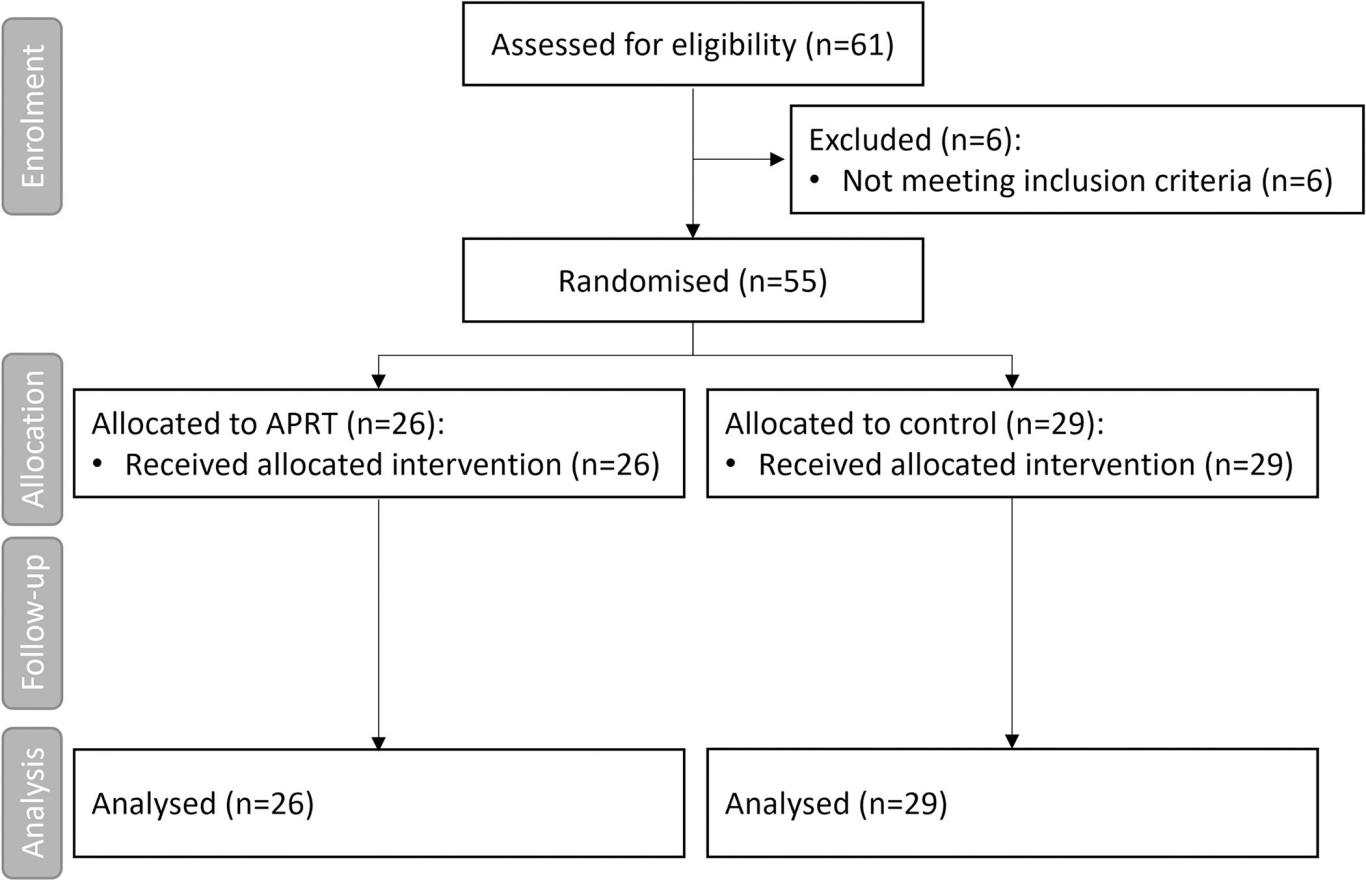
Flow diagram of study participants according to CONSORT criteria. APRT, Abbreviated Progressive Relaxation Training; n, sample.

Sixty-one volunteers were recruited for the study. Six participants were excluded for not meeting the inclusion criteria (Fig 1). The final sample consisted of 55 young participants (mean (M ± SD) = 20.3 ± 2.8; 40 females and 15 males) randomized to the relaxation (n = 26) or control (n = 29) groups. All participants had normal or corrected vision and hearing and scored above the 75^th^ percentile for trait anxiety on the STAI [41]. The STAI is a valid and reliable instrument for the assessment of state and trait anxiety. It consists of 20 items for each anxiety component and higher scores on the test represent higher levels of anxiety [41]. As reflected in the initial participation questionnaire, none of the participants were familiar with MI assessment and use. Exclusion criteria included: having suffered any physical trauma in the last 6 months; presenting visual, motor, or left-right distinction problems; history of neurological, cardiovascular, or myopathic disease; skin injury in the zones of physiological measurement devices used during the experiment; regular medication intake interfering with the nervous system.

Sample size was estimated based on a 95% confidence level (α = 0.05), a variability of the estimated parameters of 10%, a confidence interval width of 5%, and a dropout rate of 20%. The minimum sample size per group was set at 20 participants. In addition, a priori study power analysis was performed with the free program G*Power version 3.1.9.7. Finally, a total sample of 55 participants was established, which corresponded to a study power (1-β) of 0.95. Participants were recruited from the University of Alcalá and the Complutense University of Madrid, located in the Community of Madrid, Spain, through non-probabilistic consecutive sampling from 14 September 2021 to 15 September 2022. All participants attended a single session, where they volunteered to participate in the experiment after reading the Information Sheet and signing the written Informed Consent in accordance with the Declaration of Helsinki.

### Materials and variables

The primary outcome measures were four: MI capacity on the IVI, EVI, and KI subscales, global MI capacity, discrepancy time between execution and imagination times on the IVI, EVI, and KI subscales, and global discrepancy time between execution and imagination times.

These four variables were assessed using the Spanish version of the Movement Imagery Questionnaire-3 (MIQ-3) [7, 10, 42]. The MIQ-3 consists of 12 items divided into 3 subscales measuring IVI, EVI, and KI capacities, respectively. In each subscale, the same 4 movements are repeated: knee flexion and extension from a standing position, jump, horizontal adduction of the upper limb from horizontal abduction, and bilateral hip flexion and extension from a standing position. After physically performing each item, participants imagine the same movement in the required subscale and rate the ease of imagining it on a 7-point Likert scale, where 1 =“very hard to see/feel”, and 7 =“very easy to see/feel”. Higher scores represent greater MI capacity. The Spanish version of the MIQ-3 is valid and reliable for young adults. It has good structural and convergent validity, showing a Spearman correlation coefficient of 0.888 for the total questionnaire. It has also shown good levels of internal consistency and test-retest reliability for the total questionnaire, with a Cronbach’s alpha of 0.900 and an intraclass correlation coefficient of 0.866 [42].

On the other hand, TC refers to the temporal course of action [1]. Williams et al. [7] suggested that the MIQ-3 was also a good candidate for assessing TC, so this measure was added to the initial study protocol for a comprehensive assessment of imagery capacity. For this purpose, the experimenter recorded the duration of each movement’s execution and imagination with a stopwatch. The discrepancy variable was calculated as the absolute value of the difference between execution and imagination times. Lower discrepancy values represent higher TC.

Secondary outcome measures were four: the tonic component of electrodermal activity (EDA), high and low frequency bands of heart rate variability (HRV), and low and high-frequency bands ratio of HRV. These variables were used to analyze activation changes in the autonomic nervous system during relaxation and control interventions.

EDA and HRV were measured using the Empatica E4 wrist-worn device (Empatica, Milan, Italy). The E4 device is reliable and can discriminate emotional states by investigating these parameters [43].

Measured as skin conductance in microsiemens, EDA was recorded at a 4 Hz sampling frequency. Continuous decomposition analysis of the signal into tonic and phasic components was performed [44] using Ledalab 3.4.9 Free Software executed in Matlab 2022b (Mathworks®, Natick, Massachusetts, USA). The tonic component represents changes over a specific period, and the phasic component represents transient, rapid events [44]. The tonic component of the skin conductance signal was used to evaluate EDA as the dependent variable. Higher values indicated greater sympathetic system activation [45].

Blood volume pulse data necessary for HRV calculation were collected using photoplethysmography at a 64 Hz sampling frequency. Data were analyzed in the frequency domain using Kubios HRV Premium 3.5.0 software as recommended by Empatica. To obtain comparable measures among participants [46], the relative estimated power in normalized units (nu) of the low-frequency (LF) (0.04–0.15 Hz) and high-frequency (HF) (0.15–0.4 Hz) bands was calculated, as well as the LF/HF ratio [46]. LF is mainly associated with the sympathetic system, and HF with the parasympathetic system, with high LF/HF ratios indicating sympathetic dominance and low ratios indicating parasympathetic dominance [46]. The relative estimated power of LF and HF and the LF/HF ratio were used as dependent variables.

### Relaxation induction–Abbreviated Progressive Relaxation Training (APRT)

Participants in the relaxation group underwent APRT, the abbreviated version of Jacobson’s Progressive Muscle Relaxation [25]. This active relaxation technique involves consciously tensing and relaxing various muscle groups sequentially for 20 minutes. Despite its brevity, this relaxation technique effectively reduces anxiety [25–27], by eliciting physiological and psychological relaxation [27]. The relaxation instructions followed Bernstein et al. [25] guidelines and were provided via audio to standardize the intervention among participants.

### Procedure

Participants were instructed not to ingest medications or substances such as caffeine or alcohol that were likely to interfere with the nervous system in the 8 hours before the experimental session.

During the experimental session, after recording the experiment start time, the E4 device was placed on participants’ non-dominant hand following manufacturer placement protocols. The experimenter provided basic, standardized instructions on MI types. The experimenter demonstrated an example of the imagination task to ensure participants understood it but did not practice it. All participants then completed the MIQ-3 for the first time (t1) while the experimenter recorded execution and imagination times with a stopwatch at participants’ ’start’ and ’stop’ commands for each item. The relaxation group then performed APRT guided by audio, while the control group listened to neutral audio content related to the sport’s history. All participants then completed the MIQ-3 for the second time (t2) while the experimenter recorded execution and imagination times. The E4 device was then removed. The device recorded physiological variables after the first MIQ-3 (post t1) and before the second MIQ-3 (pre t2) in both groups.

### Statistical analysis

All statistical analyses were performed using RStudio software (v2022.02.3; R Core Team 2022).

Homogeneity of groups for qualitative variables was assessed using *χ^2^* tests. For quantitative variables not fitting normal distribution, Mann-Whitney U tests were performed. For those fitting normal distribution and meeting homoscedasticity principle, independent samples t-tests were performed. All hours and minutes were transformed into hourly format to analyze the homogeneity between groups concerning the time of day. Logarithmic Box-Cox transformations were performed on the primary outcome variables to allow for parametric tests. No transformations were performed for socio-demographic characteristics.

Relaxation induction effects were explored using mixed factorial ANOVA for the dependent variables EDA, LF, HF, and LF/HF ratio. Logarithmic transformations were previously applied to the EDA and LF/HF ratio. The within-subject factor PERIOD (Post t1, Pre t2) and between-subject factor GROUP (control, relaxation) were analyzed.

Group differences on MIQ-3 scores and discrepancy times were analyzed using mixed factorial ANOVAs. MIQ-3 scores and discrepancy times were log-transformed before the ANOVAs to apply this parametric statistical test. Subscale analyses included within-subject factors SUBSCALE (IVI, EVI, KI) and MOMENT (t1, t2) and the between-subject factor GROUP (control, relaxation). Global analyses included within-subject factor MOMENT (t1, t2) and between-subject factor GROUP (control, relaxation). Type I error was set at α = 5%, and eta squared (*η^2^*) effect sizes were calculated. Effect sizes were interpreted as small (*η^2^* = 0.01), medium (*η^2^* = 0.06), and large (*η^2^* = 0.14) [47]. Significant results underwent post-hoc t-tests with Bonferroni correction, and Cohen’s *d* effect sizes were calculated, interpreted as small (*d* = 0.2), medium (*d* = 0.5), and large (*d* = 0.8).

## Results

### Sample characteristics and group homogeneity

Groups were homogeneous. The experiment was conducted with no difference in the time of day between the control group (12:00h ± 2:38h) and the relaxation group (13:00h ± 2:28h) (p = 0.14). Baseline comparisons showed no differences between groups, being equivalent at t1 in MIQ-3 scores and discrepancy times (Table 1).

**Table 1:** Sample characteristics and homogeneity of the groups (control vs. relaxation).

	Control (n = 29)	Relaxation (n = 26)	Statistical	*p*-value
**Sex** [n (%)]			*χ* *^2^*	0.51
*Female*	20 (68.97%)	20 (76.92%)		
*Male*	9 (31.03%)	6 (23.08%)		
**Age ^a^**	19 (18; 21)	20 (19; 21.8)	Mann-Whitney U	0.16
**STAI- Trait (%) ^a^**	90 (85; 96)	87 (80; 90)	Mann-Whitney U	0.06
**MIQ-3 score–t1 ^b^**				
*IVI*	5.44 ± 0.96	5.27 ± 1.13	t-test	0.55
*EVI*	5.72 ± 0.88	5.19 ± 1.33	t-test	0.1
*KI*	4.85 ± 1.31	4.47 ± 1.17	t-test	0.26
*Global*	5.34 ± 1.11	4.98 ± 1.25	t-test	0.08
**Discrepancy time (s)–t1 ^b^**				
*IVI*	1.33 ± 0.88	1.80 ± 1.53	t-test	0.36
*EVI*	1.92 ± 1.26	1.95 ± 1.64	t-test	0.81
*KI*	1.86 ± 1.24	1.57 ± 1.12	t-test	0.2
*Global*	1.70 ± 1.15	1.77 ± 1.44	t-test	0.74

^a^ Values expressed as Median (Q_1_; Q_3_)

^b^ Values expressed as Mean ± SD. Abbreviation: CI = Confidence Interval; EVI = External visual Imagery; IVI = Internal visual Imagery; KI = Kinesthetic imagery; MIQ-3 = Movement Imagery Questionnaire-3; n = sample; s = second; STAI = State-Trait Anxiety Inventory; t = moment.

### Effect of relaxation

EDA was analyzed in 25 participants from the relaxation group and 26 from the control group (92.73% of participants) and HRV in 24 participants from the relaxation group and 23 from the control group (85.46% of participants).

The results for EDA, LF, HF, and LF/HF ratio showed a significant main effect of the period and a significant group × period interaction, but no significant group effect (Table 2).

**Table 2:** Results of the mixed factorial ANOVA for EDA, LF, HF and LF/FH ratio.

	Dfn	Dfd	F-value	p-value	η^2^
**EDA**					
*Group*	1	49	0.09	0.77	0.002
*Period*	1	49	46.06	<0.001***	0.062
*Group x Period*	1	49	34.75	<0.001***	0.047
**LF**					
*Group*	1	45	0.234	0.63	0.004
*Period*	1	45	4.488	0.04*	0.027
*Group x Period*	1	45	8.523	0.005**	0.049
**HF**					
*Group*	1	45	0.229	0.64	0.004
*Period*	1	45	4.525	0.039*	0.027
*Group x Period*	1	45	8.583	0.005**	0.05
**LF/HF ratio**					
*Group*	1	45	0.148	0.7	0.002
*Period*	1	45	5.11	0.029*	0.03
*Group x Period*	1	45	7.583	0.008**	0.043

Abbreviation: Dfn = Degrees of freedom in the numerator; Dfd = Degrees of freedom in the denominator; EDA = Electrodermal Activity; HF = High Frequency band; LF = Low Frequency band.

**Fig 2:**
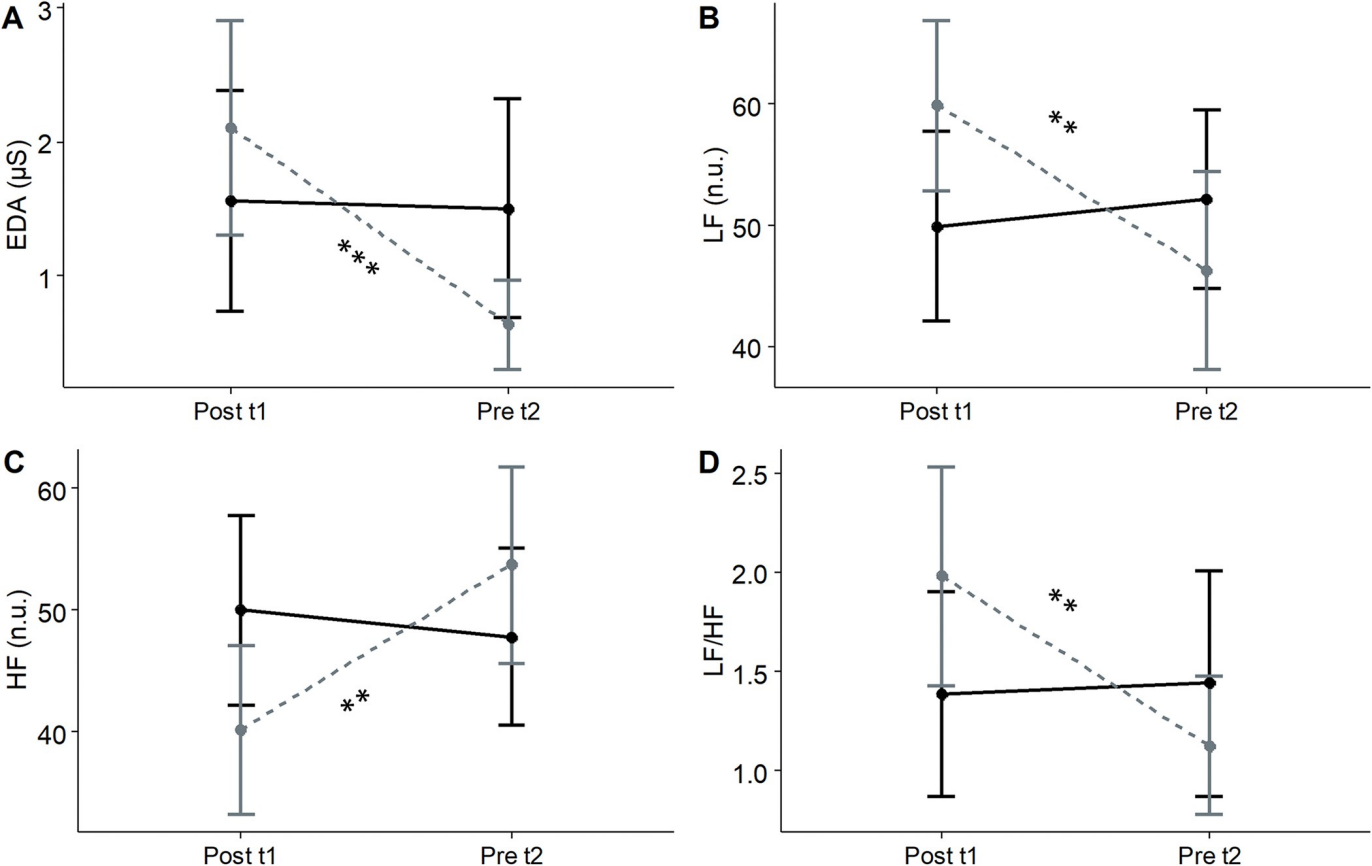
Interaction between GROUP (solid black line = control group; dashed gray line = relaxation group) and PERIOD (Post t1; Pre t2) for: A) Tonic Electrodermal Activity (EDA); B) Low frequency (LF); C) High frequency (HF); D) Low frequency/High frequency ratio (LF/HF). Error bars represent 95% confidence intervals. Significant differences with p<0.01 represented by two asterisks (**) and significant differences with p<0.001 represented by three asterisks (***). μS, microSiemens; n.u., normalized units.

Post-hoc comparisons revealed significant differences for EDA within the relaxation group between post t1 (M = 2.10, SD = 1.94) and pre t2 (M = 0.63, SD = 0.8), t(24) = 6.64, p<0.001, d = 1.33 (Fig 2A). Significant differences were also found within the relaxation group for LF between post t1 (M = 59.8, SD = 17.3) and pre t2 (M = 46.2, SD = 19.2), t(23) = 3.45, p = 0.002, d = 0.7, and for HF between post t1 (M = 40.1, SD = 17.3) and pre t2 (M = 53.7, SD = 19.2), t(23) = -3.46, p = 0.002, d = -0.71. Similarly, post-hoc comparisons revealed significant differences in the LF/HF ratio within the relaxation group between post t1 (M = 1.98, SD = 1.37) and pre t2 (M = 1.13, SD = 0.83), t(23) = 3.53, p = 0.0018, d = 0.72 (Fig 2B–2D).

### MI capacity

**Fig 3:**
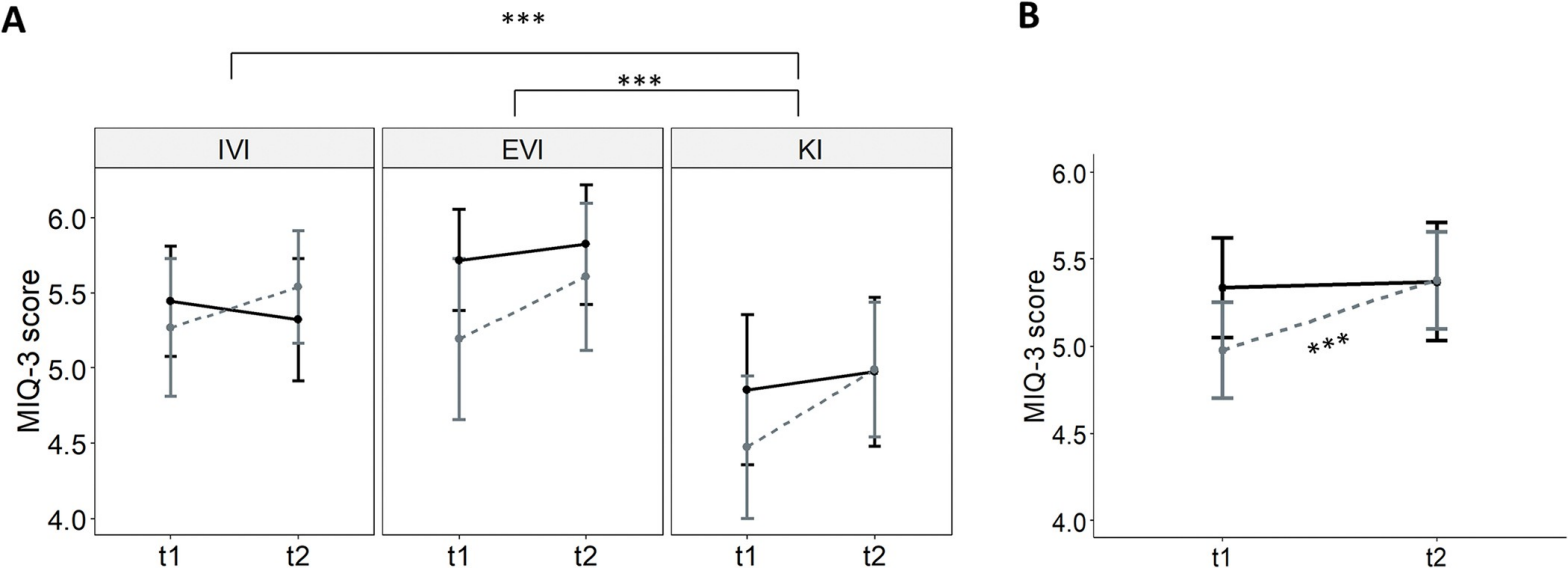
Results graph for MIQ-3 scores concerning: A) SUBSCALE factor and B) MOMENT factor. Error bars represent 95% confidence intervals. Significant differences with p<0.01 are represented by two asterisks (**), and with p<0.001 by 3 asterisks (***). The solid black line represents the control group, and the dashed gray line represents the relaxation group. EVI = external visual imagery; IVI = internal visual imagery; KI = kinesthetic imagery; t = moment.

The results for MIQ-3 subscale scores showed a significant main effect of subscale, moment, and a significant group × moment interaction (Table 3). Post-hoc comparisons revealed significant differences between the IVI subscale (M = 5.39, SD = 1.01) and the KI subscale (M = 4.83, SD = 1.23), t(195.78) = 3.77, p<0.001, d = 0.51, and significant differences between the EVI subscale (M = 5.59, SD = 1.13) and the KI subscale, t(205.53) = 4.58, p<0.001, d = 0.62, as well as significant differences within the relaxation group between t1 (M = 4.98, SD = 1.25) and t2 (M = 5.38, SD = 1.11), t(77) = -5.51, p<0.001, d = -0.62 (Fig 3).

**Table 3:** Results of the mixed factorial ANOVA for the MI ability.

	Dfn	Dfd	F-value	p-value	η^2^
**MIQ-3 scores by subscales**					
*Group*	1	159	1.038	0.31	0.006
*Subscale*	2	159	7.318	<0.001***	0.07
*Moment*	1	159	17.675	<0.001***	0.01
*Group x Subscale*	2	159	0.513	0.6	0.006
*Group x Moment*	1	159	13.636	<0.001***	0.008
*Subscale x Moment*	2	159	2.34	0.1	0.003
*Group x Subscale x Moment*	2	159	0.038	0.96	<0.001
**MIQ-3 total score**					
*Group*	1	53	0.61	0.44	0.01
*Moment*	1	53	12.45	<0.001***	0.019
*Group x Moment*	1	53	10.84	0.002**	0.016
**Discrepancy times for MIQ-3 subscales**					
*Group*	1	41	0.07	0.79	0.001
*Subscale*	2	82	2.58	0.082	0.007
*Moment*	1	41	6.23	0.017*	0.016
*Group x Subscale*	2	82	2.51	0.087	0.007
*Group x Moment*	1	41	0.498	0.484	0.001
*Subscale x Moment*	2	82	0.883	0.42	0.003
*Group x Subscale x Moment*	2	82	1.376	0.258	0.004
**Global discrepancy times for MIQ-3 items**					
*Group*	1	41	0.005	0.946	<0.001
*Moment*	1	41	6.57	0.014*	0.02
*Group x Moment*	1	41	0.366	0.548	0.001

Abbreviation: Dfn = Degrees of freedom in the numerator; Dfd = Degrees of freedom in the denominator; MIQ-3 = Movement Imagery Questionnaire-3.

The analysis for the total MIQ-3 score showed a significant main effect of moment, and a significant group × moment interaction (Table 3). Post-hoc comparisons revealed significant differences within the relaxation group between t1 and t2, t(25) = 5.1, p<0.001, d = 0.99.

### Temporal congruence

**Fig 4:**
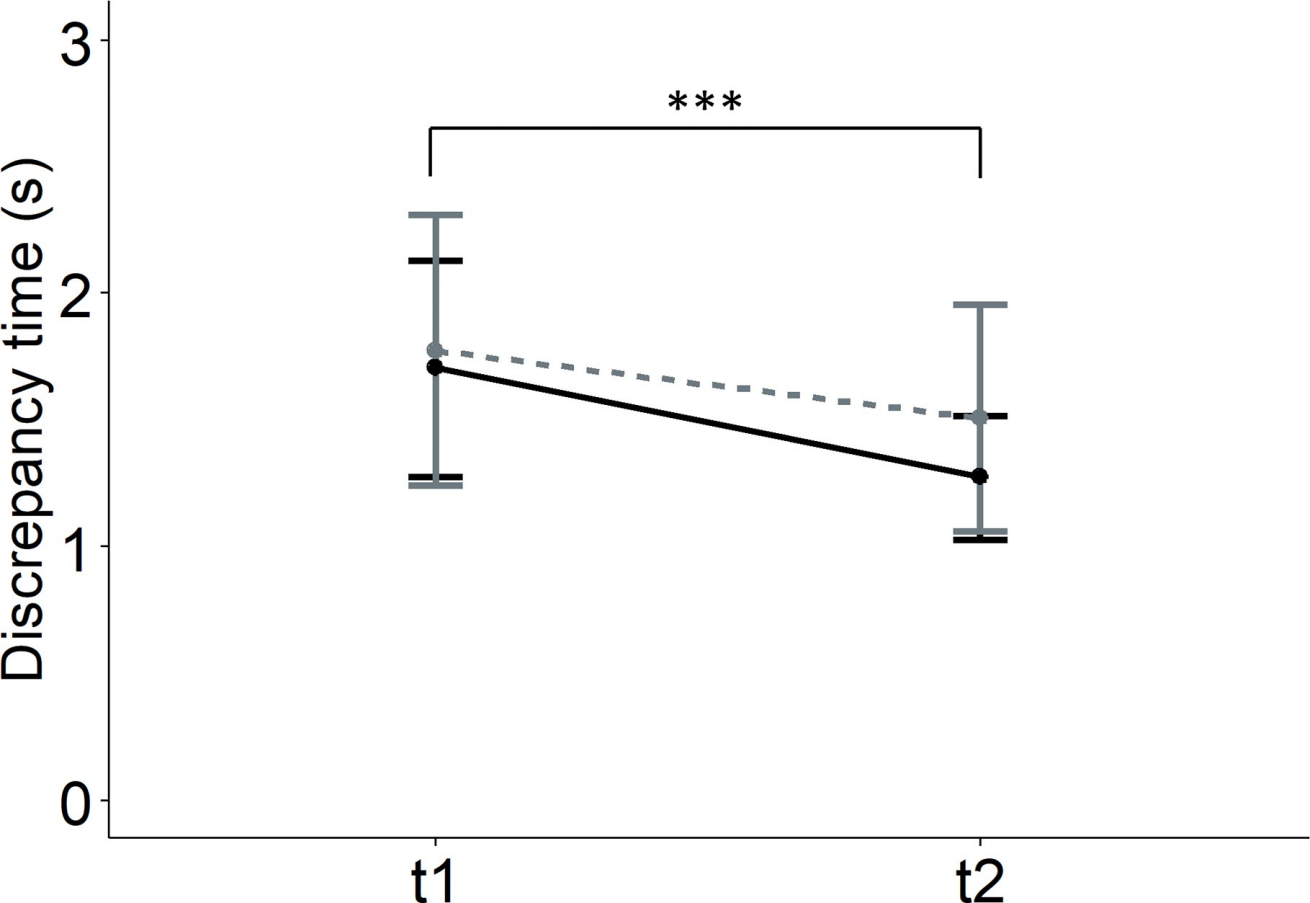
Results graph for the discrepancy time between imagination and movement execution times concerning the MOMENT factor. Error bars represent 95% confidence intervals. Significant differences with *p*<0.05 are represented by one asterisk (*). The solid black line represents the control group, and the dashed gray line represents the relaxation group. t = moment.

Temporal congruence was analyzed in forty-three participants. The results for discrepancy times by MIQ-3 subscales showed a significant main effect of moment (Table 3). Post-hoc comparisons revealed significant differences between t1 (M = 1.74, SD = 1.32) and t2 (M = 1.42, SD = 0.99), t(128) = 3.69, p<0.001, d = 0.33. The analysis for global MIQ-3 item discrepancy times also showed a main effect of moment (Table 3). Post-hoc comparisons revealed significant differences between t1 and t2, t(42) = 2.51, p = 0.016, d = 0.38 (Fig 4).

## Discussion

This study aimed to determine whether muscle relaxation before motor imagery (MI) was likely to modulate MI capacity and temporal congruence (TC) in the subscales of internal visual imagery (IVI), external visual imagery (EVI), and kinesthetic imagery (KI) in anxious individuals. It was postulated that anxious participants undergoing muscle relaxation would have better MI capacity and TC between movement execution and imagination. As expected, participants who performed abbreviated progressive relaxation training (APRT) showed better MI capacity than those not subjected to muscle relaxation across all subscales. No significant differences in TC were observed between groups, hence providing evidence that the ability to preserve the timing of the actual movement was not improved, but also not impaired.

Data first confirmed that pre-MI muscle relaxation contributed to improving MI ability in anxious individuals, hence compensating for the detrimental effects of anxiety on MI [1, 23]. As postulated by early imagery experiments in athletes, MI certainly contributed to removing external distractions and reducing somatic tensions [29–32]. APRT thereby helped participants to reach an optimal mental state for engaging in MI and form more accurate and vivid mental images. Anxious individuals are more prone to intrusive and negative thoughts as well as distractions [48], which can hinder their ability to focus on the MI task. Pre-MI relaxation might thus allow them to clear their minds of such extraneous thoughts, allowing for a more focused, immersive, and effective imagery experience. The improvement in MI capacity in the relaxation group somewhat challenges the study by Kurtz [40], who did not report any difference between performing or not performing mindfulness before MI on imagery vividness, either based on high or low anxiety levels. The difference between the two studies could be due to the characteristics of the pre-MI techniques. While mindfulness and APRT demonstrated anxiety reduction and relaxation responses [49], they remain two different approaches. Mindfulness involves accepting the present moment, without any judgment or aim, and does not target a reduction of the level of arousal [49]. In contrast, APRT aims to modify psychological and physiological states by substantially reducing physical tension and arousal level, using proprioceptive muscle stimulation [25, 27, 49]. Proprioceptive information is important for the mental representation of the body in space [50]. In this study, APRT may thus have strategically facilitated movement imagination through muscle relaxation. Data therefore suggest that APRT could be more relevant for promoting the use of accurate MI through both KI and visual imagery, either IVI or EVI. Another difference between Kurtz’s study [40] and the present work comes from the duration of the pre-MI exercise. Following Bernstein et al.’s recommendations [25], the APRT in the present study spent over 20 minutes, ensuring that the relaxation response was correctly induced in all participants as shown by EDA and HRV. In contrast, in the study by Kurtz [40], participants were subjected to a mindfulness exercise for 5 minutes, with the aim of observing the present moment without specifically targeting a relaxed state.

To our knowledge, this is the first study investigating the effectiveness of pre-MI relaxation in anxious individuals. All participants had high anxiety scores (percentile ≥ 75% on the STAI) [41], which may contribute to explaining the positive influence of pre-MI relaxation. Indeed, Louis et al. [38] found no differences in MI vividness under arousal, relaxation, or baseline conditions, but they did not assess participants’ anxiety levels. Similarly, Kurtz [40] did not specify participants’ anxiety levels. Although further studies in different samples of individuals are required before drawing firm conclusions, we postulate that pre-MI muscle relaxation may be beneficial in highly anxious individuals. Based on these results, we therefore recommend the use of pre-MI relaxation, most especially using APRT, by anxious individuals in sports, educational, or rehabilitation contexts. Additionally, given that Louis et al. [38] and Kurtz [40] did not use APRT, future studies should explore whether muscle relaxation prior to MI practice could be equally effective in improving MI ability in non-anxious individuals.

The ability of participants to reach TC between imagination and movement times improved in all groups. The fact that TC was not impaired by pre-MI relaxation is an important finding, as it prevents from harmful effects of inappropriate MI on subsequent motor performance [39]. At first sight, this finding contrasts with Louis et al.’s study [38], where relaxation before MI practice decreased TC and, therefore, MI accuracy [51]. As mentioned earlier, this may be explained by the fact that Louis et al. [38] did not include anxious participants. In this vein, we shall consider that relaxation before MI practice might negatively affect TC between imagination and movement execution in non-anxious individuals but have a more positive impact in anxious individuals, even though relaxation did not here provide additional benefits compared to the control group. This lack of group effect may be, at least partially, due to the means of the imagery time recordings. Participants indicated movement start and end by verbally communicating ’start’ and ’stop’. According to Guillot et al. [51], using movement-related signals providing temporal structure can improve the capacity to rehearse the tempo and imagine the movement in real-time. Signaling task start and end may thus have helped participants to improve TC between executed and imagined movement, regardless of APRT before MI. Additionally, the experimental design in the study by Louis et al. [38] separated actual practice from MI trials. Participants first physically performed the task, before engaging subsequently in an MI session either in a relaxed, neutral, or aroused condition. In the present study, participants in the relaxation group underwent a session of APRT before alternating physical and MI throughout all trials of the MIQ-3. Combining physical and MI may thus have improved TC regardless of the session of APRT performed before MI, as suggested by previous studies reporting greater TC when mental repetitions alternate with physical repetitions [52].

A limitation of the study lies in the devices used to record variables, as well as the complexity of some of the variables recorded. Both issues may have an impact on obtaining valid measures for the analysis.

This study was conducted on young people of both sexes with anxiety from the general population. Hence the results indicate that this entire population could benefit from the practice of muscle relaxation prior to MI in the different contexts in which MI is a technique used to improve motor learning and performance, such as rehabilitation processes and sports settings. Future studies should also explore the effect of pre-MI APRT practice in populations of different ages.

## Conclusion

The present study investigated, for the first time, whether pre-MI muscle relaxation was likely to affect internal visual, external visual, and kinesthetic imagery capacities as well as temporal congruence between imagination and movement execution in anxious individuals. Data revealed that anxious individuals substantially improved their MI capacity in each imagery modality when muscle relaxation through APRT was performed before engaging in MI. While pre-MI muscle relaxation did not provide additional benefits for improving TC between imagined and executed movement, it contributed to prevent from any impairment possibly resulting in inappropriate MI, which have been previously reported in non-anxious individuals [38]. This study thus provides a starting point to explore the beneficial effects of pre-MI muscle relaxation in anxious individuals, allowing for more effective MI programs for these individuals. Determining whether pre-MI relaxation might improve not only MI ability, but also MI effectiveness and subsequent motor performance in anxious individuals, will be an exciting focus of research in the coming years.

## Supporting information

S1 Checklist CONSORT 2010 checklist of information to include when reporting a randomised trial*. (DOC)

S1 File (PDF)

S2 File (PDF)

S1 Data (CSV)
