# Supplementary material for: Muscle relaxation enhances motor imagery capacity in people with anxiety: A randomized clinical trial
Source: PLoS One. 2025 Jan 10;20(1):e0316723. doi: 10.1371/journal.pone.0316723 (PMC11723612; doi:10.1371/journal.pone.0316723)
Supplement: S1 File — (PDF) [file pone.0316723.s002.pdf]

Protocolo de estudio: “La relajación muscular mejora la capacidad de imaginación motora en personas con ansiedad: un ensayo clínico aleatorizado”

Esta investigación pertenece al proyecto de tesis doctoral ***“Influencia de los estados de alerta, estrés y ansiedad en el aprendizaje motor de una tarea manual precisa y en la capacidad de imaginación motora”*****,** que cuenta con la aprobación del Comité de Ética de la Investigación y de Experimentación Animal de la Universidad de Alcalá con código CEID2022/2/036. Dicho proyecto de tesis engloba tres grandes estudios. Esta investigación forma parte del estudio: “***Influencia de la ansiedad en el aprendizaje motor de una tarea manual precisa, en la memoria a corto y largo plazo y en la capacidad de imaginación motora***”, que fue registrado en ClinicalTrails.gov con código NCT04973956. A continuación, se expone el protocolo de estudio aprobado por el Comité de Ética, con los datos extraídos relativos a la investigación: “***La relajación muscular mejora la capacidad de imaginación motora en personas con ansiedad: un ensayo clínico aleatorizado***”.

1. Hipótesis de trabajo:

- Los sujetos con ansiedad, a los cuales se les induzca relajación, serán los que muestren una mejor capacidad de imaginación motora.
- Los sujetos con ansiedad, a los cuales no se les induzca relajación, serán los que muestren una peor capacidad de imaginación motora.

2. Objetivo:

- Conocer si la capacidad de imaginación visual interna, visual externa y cinestésica varía cuando los participantes con ansiedad se someten a relajación.

3. Metodología:

El desarrollo del estudio tendrá lugar en una sala acondicionada al efecto perteneciente al Departamento de Fisioterapia de la Facultad de Enfermería y Fisioterapia de la Universidad de Alcalá, y en la Facultad de Psicología de la Universidad Complutense de Madrid. El estudio también podrá llevarse a cabo en instalaciones de otros centros universitarios españoles e internacionales en los que la doctoranda pueda desarrollar parte de su proyecto de investigación, previo consentimiento y permisos correspondientes de los respectivos centros.

- **Diseño:**

  Estudio longitudinal, experimental, aleatorio y a doble ciego (el sujeto que realiza la prueba no sabe qué hacen los otros sujetos, un investigador ciego a la intervención analizará los datos para obtener los resultados) (1).

  La aleatorización se llevará a cabo a través de asignación estratificada mediante el estrato sexo (masculino o femenino), para controlar que la proporción de hombres y mujeres sea homogénea en todos los grupos. Dentro de cada estrato se realizará asignación aleatoria simple a través de sobres cerrados, que el participante facilitará al investigador en el momento del estudio con el objetivo de mantener la ocultación de la asignación, evitando así un posible sesgo de selección (1).

- **Participantes:**

  La población de estudio estará compuesta por sujetos que estén cursando estudios de grado y postgrado en la Universidad de Alcalá y en la Universidad Complutense de Madrid, así como, en caso de que la situación lo permita y se puedan realizar estancias de investigación en otros centros, por sujetos que pertenezcan a otros centros universitarios españoles e internacionales en los que la doctoranda pueda desarrollar parte de su proyecto de investigación. Todos los sujetos incluidos en el estudio deberán cumplir los criterios de selección correspondientes y participar de manera voluntaria en el mismo tras la lectura de la hoja informativa y firma del consentimiento informado.

  ✓ **Criterios de inclusión:**

    - Hombres y mujeres entre 18 y 35 años.
    - Visión normal o corregida especificando el método de corrección.
    - Audición normal o corregida especificando el método de corrección.
    - No familiarizados con la valoración y usos de la imaginación motora.
    - Presencia de puntuaciones en el STAI correspondientes con ansiedad.

  × **Criterios de exclusión:**

    - Sujetos que hayan sufrido fracturas, luxaciones o procesos traumáticos en cualquier segmento del miembro superior no dominante o de dedos, muñeca o codo del miembro superior dominante en los últimos 6 meses.
    - Sujetos con trastornos del aprendizaje o problemas para leer o escribir.
    - Sujetos con historia de cualquier enfermedad neurológica, cardiovascular, miopática, crisis epiléptica, crisis de ausencia, apnea del sueño, síndrome de fatiga crónica o fibromialgia.

- Estar tomando alguna medicación para suprimir la ansiedad, para dormir, antidepresivos, antihistamínicos, relajantes musculares, psicotrópica u otras que interfieran sobre el sistema nervioso.
- Toma de sustancias depresoras o estimulantes del sistema nervioso como cafeína o teína en las últimas 8 horas.

- **Determinación del tamaño muestral:**

  Debido a las características novedosas del estudio, el marco muestral es desconocido. Dado que no se han encontraron estudios previos de referencia para hacer el cálculo del tamaño muestral, se ha estimado un tamaño muestral *n*, en función del nivel de confianza Z_α, la varianza estimada *s* y la precisión d. Donde con un nivel de confianza del 95% (Z_α =1.96), una varianza estimada del 10% y una precisión del 5%:

$$n = \frac{Z\alpha^2 s^2}{d^2} = \frac{1.96^2\ 0.1^2}{0.05^2} = 15.3664$$

Ecuación 1. Cálculo tamaño muestral

El tamaño muestral final *N* se ha obtenido estimando una proporción de pérdidas *R* del 20%:

$$N = n\frac{1}{1-R} = 15.3664\frac{1}{1-0.20} = 19{,}208$$

Ecuación 2. Corrección de la muestra por pérdidas

Por lo tanto, se ha estimado que en cada grupo de intervención deberá reclutarse un mínimo de 20 sujetos para cumplir las condiciones antedichas.

El muestreo será no probabilístico consecutivo. No intervendrá el azar (probabilístico) y se seleccionará a los sujetos que cumplan los criterios de selección a medida que vayan aceptando participar en el mismo (consecutivo) (1).

- **Instrumentos de medida:**

  - ***State-Trait Anxiety Inventory* (STAI):** Es uno de los instrumentos más utilizados para la evaluación auto-informada de la ansiedad (2,3). Hay varias versiones del cuestionario y para esta tesis se utilizará la adaptación española de 40 ítems, con 20 ítems dedicados a cada subescala (2,3). Cada ítem se puntúa en una escala Likert de 4 puntos que va desde el 0=casi nunca/nada hasta el 3=mucho/casi siempre. La puntuación total de cada subescala va de 0 a 60 con valores más altos representando mayores niveles de ansiedad. Esta versión ha

demostrado buenas propiedades psicométricas en universitarios españoles, con valores de consistencia interna mayores de 0.80, alfa de Cronbach de 0.93 y buenos valores de validez interna.

- ***Movement Imagery Questionnaire-3*** **(MIQ-3):** Es la versión más reciente del *Movement Imagery Questionnaire-Revised* (MIQ-R). Consta de 12 ítems repartidos en 3 subescalas, que miden la capacidad de imaginar cinestésica, visual interna y visual externa. Así, en cada una de las 3 subescalas se repiten los mismos 4 movimientos: salto, flexionar una pierna, movimiento de brazo desde el lado hacia el frente y flexión de cadera. Tras ejecutar físicamente cada ítem, los participantes deben imaginar el movimiento en la subescala requerida y puntuar la facilidad o dificultad para realizar el movimiento en una escala de Likert de 7 puntos, dónde 1=muy difícil de ver/sentir el movimiento y 7=muy fácil de ver/sentir el movimiento (4). Por tanto, mayores puntuaciones representan mayor capacidad de imaginación. Para el desarrollo de esta tesis se utilizará su versión española.

- **Medidas fisiológicas:** Para confirmar que se ha generado un efecto emocional significativo, se registrarán datos fisiológicos en tiempo real a través del dispositivo de muñeca Empatica E4 (Empatica, Milán, Italia) (5). Este dispositivo cumple la certificación necesaria de la Comisión Europea para dispositivos médicos (5) y a través de él se medirá:
  - → **Actividad electrodérmica de la piel (AED):** Es una medida indirecta no invasiva de la activación del Sistema Nervioso Autónomo (SNA) (6). Mide las características bioeléctricas de la piel aplicando una corriente directa en la misma y registrando el resultado como conductancia cutánea (7). El dispositivo E4 muestreará la actividad electrodérmica a una frecuencia de muestreo de 4 Hz (5) a través de dos electrodos de plata.
  - → **Variabilidad de la frecuencia cardiaca (VFC):** Es un fenómeno fisiológico de variación en el intervalo de tiempo entre latidos (R-R) y también es utilizado comúnmente como medida de la actividad del SNA (6). El dispositivo E4 medirá el pulso del volumen sanguíneo a una frecuencia de muestreo de 64 Hz a través de sus sensores de fotopletismografía. A partir de esta medida se derivará la variabilidad del ritmo cardíaco (5).

  Los datos obtenidos con el dispositivo E4 se descargarán y guardarán en formato .csv a través del software Empatica para su posterior análisis (5).

- **Intervención: Relajación Muscular Progresiva Abreviada (RMPA).**
  Es una de las técnicas de relajación más utilizadas en investigación por su facilidad de implementación, bajo coste y aplicabilidad en entornos clínicos y no clínicos. Para este estudio utilizaremos su versión abreviada (8), que consiste en una sesión estandarizada de 20 min en los que se pide a los sujetos tensar y relajar secuencialmente varios grupos musculares. A pesar de su brevedad, esta técnica de relajación ha demostrado ser efectiva para reducir el estrés y la ansiedad. Así, se ha visto que produce relajación cognitiva, comportamental y fisiológica (8).

- **Variables**
  - **Variable independiente: Ansiedad.** Describirá si el sujeto con ansiedad ha sido sometido o no a intervención de relajación.
  - **Variables dependientes:**
    - **Actividad electrodérmica**.
    - **Variabilidad de la frecuencia cardiaca**.
    - **Capacidad de imaginar.** Se medirá a través del MIQ-3, diferenciando entre las subescalas de imaginación cinestésica, visual externa y visual interna.
  - **Variables control:**
    - **Género.** Se controlará que la proporción de hombres y mujeres sea homogénea en todos los grupos.
    - **Edad.** Se controlará a través de los criterios de selección que los participantes tengan entre 18 y 35 años.

- **Procedimiento y condiciones experimentales:**
  Tras la difusión de la información para participar en el estudio el procedimiento será:
  1º) Los sujetos leerán la Hoja de información y decidirán participar libremente en el estudio mediante la firma escrita del Consentimiento Informado.
  2º) Se pasará un cuestionario inicial para asegurar que los sujetos cumplen los criterios de inclusión en el estudio y se codificará mediante un número de identificación asignado al nombre del sujeto, con el que se le identificará durante el desarrollo del estudio.
  3º) Se citará a los sujetos en el lugar acondicionado al efecto en el centro correspondiente. El día previo al experimento se les recordarán las pautas que tienen que seguir de preparación para el estudio, aspectos que serán

comprobados también el mismo día del estudio para asegurar que los sujetos siguen cumpliendo los criterios de selección.

4º) Los participantes realizarán el STAI. Aquellos con puntuaciones indicativas de ansiedad serán incluidos en el estudio.

5º) Así, los participantes con ansiedad serán asignados aleatoriamente a través de sobres cerrados estratificados por sexo, y ajenos al investigador que lleve a cabo el experimento, a uno de los dos grupos experimentales: relajación (G1) o control (G2).

6º) Después se les colocará la pulsera Empatica E4 de medición de medidas fisiológicas y comenzarán a grabarse las variables fisiológicas desde ese momento.

7º) Los participantes realizarán el MIQ-3, cuyo resultado será recogido en la 'Hoja de recogida de datos'.

8º) Según el grupo al que haya sido asignado cada participante:

- **Grupo 1 (de relajación):** Se sentará a los participantes en un asiento reclinado en una sala con la luz tenue. Se realizará la sesión de 20 min de RMPA. Con la finalidad de homogeneizar las condiciones y evitar sesgos debidos al investigador, todos escucharán un audio con las instrucciones de relajación.
- **Grupo 2 (control):** Se sentará a los participantes en una silla normal, con luz normal y permanecerán los 20 min sentados mientras escuchan un audio de contenido neutro.

9º) Los participantes completarán el MIQ-3 por segunda vez.

10º) Se retirará el dispositivo E4 de medidas fisiológicas.

- **Análisis de los datos:**

El análisis y procesamiento de datos procedentes de la versión R2020b de Matlab y la pulsera Empatica E4 será realizado a través del programa estadístico SPSS 25.0. y el software Kubios HRV (para el análisis de la VFC) (9).

En primer lugar, se realizarán los análisis estadísticos adecuados para ver si los datos se distribuyen de acuerdo con una curva normal a través de la prueba de Shapiro-Wilk y para valorar la influencia del sexo y la edad.

Posteriormente, se realizará el análisis descriptivo de las frecuencias relativa y absoluta para las variables cualitativas de género y edad y se comprobará su homogeneidad mediante la prueba Chi-cuadrado. También se realizará el análisis descriptivo de las variables cuantitativas y se valorará si se ajustan a la normal.

Se realizará el análisis comparativo mediante ANOVA con respecto a la capacidad de imaginar, comparando los resultados obtenidos en los dos momentos respectivos dentro del desarrollo del experimento.
También se estudiará la actividad electrodérmica y la variabilidad de la frecuencia cardiaca durante la inducción de la relajación mediante un ANOVA.
Para los contrastes de hipótesis se fijará un nivel de significación de 0,05.

- **Aspectos éticos y legales:**

El estudio se realizará siguiendo todas las leyes, normas y recomendaciones tanto nacionales como internacionales, y deberán haber obtenido el dictamen favorable del Comité de Ética de Investigación y Experimentación Animal de la Universidad de Alcalá.
Los sujetos tendrán que dar su consentimiento expreso para participar en el proyecto mediante la firma del Consentimiento Informado, tras la lectura de la hoja informativa u otra información adicional que desee recibir por parte del equipo investigador. Se respetarán así los principios éticos para las investigaciones con seres humanos de la Declaración de Helsinki y la ley básica 41/2002 del 14 de noviembre, mediante la que se regula la Autonomía del paciente y los derechos y obligaciones en materia de información y documentación clínica.
Una vez que los sujetos entren a participar en el estudio, se aplicará la legislación vigente y aplicable con relación a sus derechos respecto a la protección de datos personales. Se actuará conforme al Reglamento General de Protección de Datos (UE) 2016/679 y la Ley Orgánica 3/2018 del 5 de diciembre, de Protección de Datos Personales y garantía de los derechos digitales. Los datos serán tratados de forma anónima en el marco de la función investigadora atribuida legalmente a la Universidad.
Así, los datos que se recojan en el estudio serán confidenciales y solo serán manejados por el equipo investigador. A través de un proceso de codificación, se asignará a los participantes un número identificativo con el que se les identificará durante el desarrollo de todo el estudio. Todos los resultados que se obtengan formarán parte de una base de datos en la que se mantendrán anónimos y solo serán manejados y custodiados por el grupo investigador con el único fin del correcto desarrollo del estudio. De ningún modo el nombre de los participantes aparecerá en ninguna publicación o informe relativo al estudio.
A través de la hoja de información, se hará saber a los sujetos que participen en el estudio que pueden ejercer una serie de derechos sobre los datos que serán recogidos para el citado estudio, sin tener que dar ninguna explicación. Estos

derechos contemplan la posibilidad de acceder a los mismos, rectificar (los datos que estén incompletos o sean erróneos), cancelar (solicitar que se bloquee su uso), limitar el tratamiento de aquellos datos que sean incorrectos, oponerse al uso de estos, solicitar una copia o que se trasladen a un tercero (portabilidad). También se les hará saber que, para ejercitar los derechos citados, pueden dirigirse al investigador principal del estudio y/o a la responsable del tratamiento de los datos, que en este caso será la Secretaría General de la Universidad de Alcalá, legitimada para ello, y ante quien se podrán ejercer los correspondientes derechos por escrito o por mail (protecciondedatos@uah.es).

Además, se solicitará el permiso correspondiente en cada Dirección del Centro Universitario nacional o internacional en el que sea necesario un permiso adicional.

4. Planificación temporal:

T3.1. Difusión y publicidad para la captación de sujetos.
T3.2. Captación de la población de estudio.
T3.3. Información a los sujetos y firma del consentimiento informado.
T3.4. Selección y aleatorización de los sujetos con asignación de códigos.
T3.5. Valoración inicial de los sujetos.
T3.6. Realización de la intervención.
T3.7. Análisis y redacción de resultados de los datos recogidos.
T3.8. Difusión de los resultados.

Tareas	Meses										
	1	2	3	4	5	6	7	8	9	10	11	12-24
T3.1.												
T3.2.												
T3.3.												
T3.4.												
T3.5.												
T3.6.												
T3.7.												
T3.8.												

5. Referencias bibliográficas:

1. Argimón Pallás JM, Jiménez Villa J. Métodos de investigación clínica y epidemiología. 3ª ed. Madrid: Elsevier España; 2004.
2. Spielberger CD, Gorsuch R, Lushene R. STAI. Cuestionario de ansiedad estado-rasgo. 7ª ed. Madrid: TEA; 2008.

3. Fonseca-Pedrero E, Paino M, Sierra-Baigrie S, Lemos-Giráldez S, Muñiz J. Propiedades psicométricas del “Cuestionario de Ansiedad Estado-Rasgo” (STAI) en universitarios. Psicol Conductual. 2012; 20 (3): 547-561.
4. Williams SE, Cumming J, Ntoumanis N, Nordin-Bates SM, Ramsey R, Hall C. Further validation and development of the Movement Imagery Questionnaire. J Sport Exerc Psychol. 2012; 34 (5): 621-46.
5. Empatica Inc. [Internet]. Milán: Empatica Inc; 2019 [consultado el día 20 May 2020]. Disponible en: https://www.empatica.com/en-eu/research/e4/
6. Bali A, Singh Jaggi A. Clinical experimental stress studies: methods and assessment. Rev Neurosci. 2015; 26(5): 555-579.
7. Noteboom JT, Fleshner M, Enoka RM. Activation of the arousal response can impair performance on a simple motor task. J App Physiol. 2001; 91: 821-831.
8. Bernstein DA, Carlson CR, Schmidt JE. Progressive relaxation: abbreviated methods. En: Lehrer PM, Woolfolk RL, Sime WE. Principles and practice of stress management. New York: The Guilford Press; 2007. 88-122.
9. Tarvainen MP, Niskanen JP, Lipponen JA, Ranta-aho PO, Karjalaien PA. Kubios HRV-Heart Rate Variability analysis software. Comput Methods Programs Biomed. 2014; 113 (1): 210- 220.
