# Supplementary material for: Muscle relaxation enhances motor imagery capacity in people with anxiety: A randomized clinical trial
Source: PLoS One. 2025 Jan 10;20(1):e0316723. doi: 10.1371/journal.pone.0316723 (PMC11723612; doi:10.1371/journal.pone.0316723)
Supplement: S2 File — (PDF) [file pone.0316723.s003.pdf]

<u>Study protocol: “Muscle relaxation enhances motor imagery capacity in people with anxiety: a randomized clinical trial”.</u>

This research belongs to the doctoral thesis project **“*Influence of arousal, stress and anxiety states on the motor learning of a precise manual task and the motor imagery capacity*”,** approved by the Ethics Committee for Research and Animal Experimentation of the University of Alcalá with code CEID2022/2/036. This thesis project encompasses three major studies. This research is part of the study: “***Influence of Anxiety on Motor Learning and Motor Imagery Ability in Young Population***”, registered in ClinicalTrails.gov with code NCT04973956. The following is the study protocol approved by the Ethics Committee, with data extracted concerning the research: “***Muscle relaxation enhances motor imagery capacity in people with anxiety: a randomized clinical trial***”.

1. **Hypothesis:**
    - Anxious participants who are induced to relax will show better motor imagery capacity.
    - Anxious participants, who are not induced to relax, will show a poorer motor imagery capacity.

2. **Objetivo:**
    - To determine whether internal visual, external visual, and kinesthetic imagery capacity varies when participants with anxiety are subjected to relaxation.

3. **Methodology:**

The study will take place in a room fitted out for this purpose, which belongs to the Department of Physiotherapy of the Faculty of Nursing and Physiotherapy of the University of Alcalá and the Faculty of Psychology of the Complutense University of Madrid. The study may also be conducted in the facilities of other Spanish and international university centers where the PhD student may carry out part of her research project, subject to the prior consent and permissions of the respective centers.

- **Design:**

  Longitudinal, experimental, randomized, double-blind study (the subject performing the test does not know what the other subjects are doing, a researcher blind to the intervention will analyze the data to obtain the results) (1).

Randomization will be done through stratified allocation by gender stratum (male or female), to ensure that the proportion of males and females is homogeneous in all groups. Within each stratum, simple random allocation will be performed through sealed envelopes, which the participant will provide to the researcher during the study to maintain allocation concealment, thus avoiding possible selection bias (1).

- **Participants:**

  The study population will be composed of subjects who are pursuing undergraduate and postgraduate studies at the University of Alcalá and the Complutense University of Madrid, as well as, if the situation permits and research stays can be carried out in other centers, subjects belonging to other Spanish and international university centers where the doctoral student can develop part of her research project. All subjects included in the study must meet the corresponding selection criteria and participate voluntarily in the study after reading the information sheet and signing the informed consent form.

  ✓ **Inclusion criteria:**

  - Men and women aged 18-35 years.
  - Normal or corrected vision specifying the method of correction.
  - Normal or corrected hearing specifying the method of correction.
  - Unfamiliar with the assessment and uses of motor imagery.
  - Presence of corresponding STAI scores with anxiety.

  × **Criterios de exclusión:**

  - Subjects who have suffered fractures, dislocations, or traumatic processes in any segment of the non-dominant upper limb or fingers, wrist, or elbow of the dominant upper limb in the last 6 months.
  - Subjects with learning disabilities or problems in reading or writing.
  - Subjects with a history of any neurological disease, cardiovascular disease, myopathic disease, epileptic seizure, absence seizure, sleep apnea, chronic fatigue syndrome, or fibromyalgia.
  - Taking any medication to suppress anxiety, to sleep, antidepressants, antihistamines, muscle relaxants, psychotropics, or other medications that interfere with the nervous system.
  - Taking nervous system depressants or stimulants such as caffeine or theine in the last 8 hours.

- **Sample size determination:**

  Due to the novel characteristics of the study, the sample frame is unknown. Since no previous reference studies have been found to calculate the sample size, a sample size n has been estimated, based on the confidence level Z_{α}, the estimated variance s, and the precision d. Where with a confidence level of 95% (Z_{α} =1.96), an estimated variance of 10%, and a precision of 5%:

$$n = \frac{Z\alpha^2 s^2}{d^2} = \frac{1.96^2\ 0.1^2}{0.05^2} = 15.3664$$

Equation 1. Sample size calculation

  The final sample size *N* has been obtained by estimating a loss ratio *R* of 20%:

$$N = n\frac{1}{1-R} = 15.3664\frac{1}{1-0.20} = 19{,}208$$

Equation 2. Correction of the sample

  Therefore, it has been estimated that a minimum of 20 subjects should be recruited in each intervention group to meet the above conditions.

  The sampling will be non-probability consecutive. It will be non-random (probability) and subjects meeting the selection criteria will be selected as they agree to participate (consecutive) (1).

- **Measuring instruments:**

  - ***State-Trait Anxiety Inventory* (STAI):** is one of the most widely used instruments for self-reported assessment of anxiety (2,3). There are several versions of the questionnaire and for this thesis, the 40-item Spanish adaptation will be used, with 20 items dedicated to each subscale (2,3). Each item is scored on a 4-point Likert scale ranging from 0=almost never/not at all to 3=very much/almost always. The total score for each subscale ranges from 0 to 60 with higher values representing higher anxiety. This version has shown good psychometric properties in Spanish university students, with internal consistency values higher than 0.80, Cronbach's alpha of 0.93, and good internal validity values.

  - ***Movement Imagery Questionnaire-3* (MIQ-3):** this is the most recent version of the *Movement Imagery Questionnaire-Revised* (MIQ-R). It consists of 12 items divided into 3 subscales, which measure kinesthetic, internal visual, and external visual imagery. Thus, in each of the 3 subscales, the same 4 movements are

repeated: jumping, leg flexion, arm movement from the side to the front, and hip flexion. After physically performing each item, participants must imagine the movement in the required subscale and rate the ease or difficulty of performing the movement on a 7-point Likert scale, where 1=very difficult to see/feel the movement and 7=very easy to see/feel the movement (4). Thus, higher scores represent greater motor imagery capacity. The Spanish version will be used.

- **Physiological measures:** To confirm that a significant emotional effect has been generated, physiological data will be recorded in real-time using the Empatica E4 wrist device (Empatica, Milan, Italy) (5). This device complies with the required European Commission certification for medical devices (5) and will be used to measure:
  - → **Electrodermal activity (EDA):** this is a non-invasive indirect measure of Autonomic Nervous System (ANS) activation (6). It measures the bioelectrical characteristics of the skin by applying a direct current to the skin and recording the result as skin conductance (7). The E4 device will sample electrodermal activity at a sampling rate of 4 Hz (5) through two silver electrodes.
  - → **Heart rate variability (HRV):** this is a physiological phenomenon of variation in the time interval between beats (R-R) and is also commonly used as a measure of ANS activity (6). The E4 device will measure the blood volume pulse at a sampling rate of 64 Hz through its photoplethysmography sensors. From this measurement, heart rate variability will be derived (5).

  The data obtained with the E4 device shall be downloaded and saved in .csv format via Empatica software for further analysis (5).

- **Intervention: Abbreviated Progressive Relaxation Training (APRT).**
  It is one of the most widely used relaxation techniques in research due to its ease of implementation, low cost, and applicability in clinical and non-clinical settings. For this study, we will use its abbreviated version (8), which consists of a standardized 20-minute session in which subjects are asked to sequentially tense and relax various muscle groups. Despite its brevity, this relaxation technique reduces stress and anxiety. It has been shown to produce cognitive, behavioral, and physiological relaxation (8).

- **Variables**
  - **Independent variable: Anxiety.** It will describe whether or not the subject with anxiety has undergone relaxation intervention.
  - **Dependent variables:**
    - **EDA**.
    - **HRV**.
    - **Motor imagery capacity.** It will be measured through the MIQ-3, differentiating between the subscales of kinesthetic, external visual, and internal visual imagery.
  - **Control variables:**
    - **Gender.** The proportion of men and women will be controlled to ensure that the proportion of men and women is homogeneous in all groups.
    - **Age.** The selection criteria will ensure that participants are between 18 and 35.

- **Procedure and experimental conditions:**

  After the dissemination of information to participate in the study the procedure will be:

  1st) Subjects will read the Information Sheet and freely decide to collaborate in the study by signing the Informed Consent form in writing.

  2nd) An initial questionnaire will be filled in to ensure that the subjects meet the criteria for inclusion in the study. Participants will be coded using an identification number assigned to the subject's name, with which they will be identified during the study.

  3rd) Subjects will be summoned to the place set up for this purpose at the corresponding center. The day before the experiment, they will be reminded of the guidelines they must follow in preparation for the study. The guidelines will also be checked on the experiment day to ensure that the subjects continue to meet the selection criteria.

  4th) Participants will take the STAI. Those with scores indicative of anxiety will be included in the study.

  5th) Thus, participants with anxiety will be randomly assigned through closed envelopes stratified by sex, and external to the researcher conducting the experiment, to one of the two experimental groups: relaxation (G1) or control (G2).

6th) Afterwards, the Empatica E4 wristband for measuring physiological measurements will be placed on them and the physiological variables will start to be recorded from that moment onwards.

7th) Participants will take the MIQ-3, the results of which will be recorded on the 'Data collection sheet'.

8th) According to the group to which each participant has been assigned:

- **Group 1 (relaxation):** participants will be seated in a reclining seat in a dimly lit room. The 20-minute APRT session will be conducted. To homogenize conditions and avoid researcher bias, everyone will listen to an audio relaxation instruction.
- **Group 2 (control):** participants will be seated in a normal chair, with normal lighting, and will remain seated for 20 min while listening to neutral audio.

9th) Participants shall complete the MIQ-3 for the second time.

10th) The E4 physiological measurement device shall be removed.

- **Data analysis:**

The analysis and processing of data coming from Matlab version R2020b and the Empatica E4 wristband will be performed through the statistical program SPSS 25.0. and the software Kubios HRV (for HRV analysis) (9).

First, appropriate statistical analyses will be performed to see if the data are distributed according to a normal curve through the Shapiro-Wilk test and to assess the influence of sex and age.

Subsequently, the descriptive analysis of the relative and absolute frequencies for the qualitative variables of gender and age will be carried out and their homogeneity will be tested using the Chi-square test. The descriptive analysis of the quantitative variables will also be carried out and their normality will be assessed.

A comparative analysis will be carried out using ANOVA concerning motor imagery capacity, comparing the results obtained at the two respective moments in the development of the experiment.

Electrodermal activity and heart rate variability during relaxation induction will also be studied using ANOVA.

A significance level of 0.05 will be set for the hypothesis tests.

- **Ethical and legal aspects:**

  The study will develop following all national and international laws, regulations, and recommendations. It must have obtained the favorable opinion of the Ethics Committee for Research and Animal Experimentation of the University of Alcalá.

  Participants will have to give their express consent to participate in the project by signing the Informed Consent form, after reading the information sheet or any other additional information they may wish to receive from the research team. The ethical principles for research with human beings of the Declaration of Helsinki and the basic law 41/2002 of 14 November, which regulates patient autonomy and the rights and obligations regarding information and clinical documentation, will thus be respected.

  Once the subjects enter to participate in the study, the current and applicable legislation will be applied to their rights regarding the protection of personal data. The General Data Protection Regulation (EU) 2016/679 and the Organic Law 3/2018 of 5 December on the Protection of Personal Data and guarantee of digital rights will be acted upon. The data will be processed anonymously within the framework of the research function legally attributed to the University.

  Thus, the data collected in the study will be confidential and handled by the research team. Through a coding process, participants will be assigned an identification number with which they will be identified throughout the study. All the results obtained will form part of a database in which they will remain anonymous and will only be handled and kept by the research team for the sole purpose of the proper conduct of the study. In no way will the names of the participants appear in any publication or report related to the study.

  Through the information sheet, the subjects participating in the study will be informed that they can exercise a series of rights over the data that will be collected for the research, without having to give any explanation. These rights include the possibility of accessing the data, rectifying (data that are incomplete or erroneous), canceling (requesting that their use be blocked), limiting the processing of incorrect data, opposing their use, requesting a copy or that they be transferred to a third party (portability). They will also be informed that to exercise these rights, they may contact the principal investigator of the study or the person responsible for the processing of the data, which in this case will be the General Secretariat of the University of Alcalá, legitimized to do so, and before whom the corresponding rights may be exercised in writing or by e-mail (protecciondedatos@uah.es).

  In addition, the corresponding permission will be requested from each national or international University Centre Directorate where additional permission is required.

4. Time planning:

T3.1. Dissemination and publicity for recruitment of subjects.

T3.2. Recruitment of the study population.

T3.3. Information to subjects and signing of informed consent.

T3.4. Selection and randomization of subjects with assignment of codes.

T3.5. Initial assessment of subjects.

T3.6. Implementation of the intervention.

T3.7. Analysis and results of the data collected.

T3.8. Dissemination of results.

**Tasks**	**Months**											
	**1**	**2**	**3**	**4**	**5**	**6**	**7**	**8**	**9**	**10**	**11**	**12-24**
T3.1.												
T3.2.												
T3.3.												
T3.4.												
T3.5.												
T3.6.												
T3.7.												
T3.8.												

5. References:

1. Argimón Pallás JM, Jiménez Villa J. Métodos de investigación clínica y epidemiología. 3ª ed. Madrid: Elsevier España; 2004.
2. Spielberger CD, Gorsuch R, Lushene R. STAI. Cuestionario de ansiedad estado-rasgo. 7ª ed. Madrid: TEA; 2008.
3. Fonseca-Pedrero E, Paino M, Sierra-Baigrie S, Lemos-Giráldez S, Muñiz J. Propiedades psicométricas del “Cuestionario de Ansiedad Estado-Rasgo” (STAI) en universitarios. Psicol Conductual. 2012; 20 (3): 547-561.
4. Williams SE, Cumming J, Ntoumanis N, Nordin-Bates SM, Ramsey R, Hall C. Further validation and development of the Movement Imagery Questionnaire. J Sport Exerc Psychol. 2012; 34 (5): 621-46.
5. Empatica Inc. [Internet]. Milán: Empatica Inc; 2019 [consultado el día 20 May 2020]. Disponible en: https://www.empatica.com/en-eu/research/e4/
6. Bali A, Singh Jaggi A. Clinical experimental stress studies: methods and assessment. Rev Neurosci. 2015; 26(5): 555-579.

7. Noteboom JT, Fleshner M, Enoka RM. Activation of the arousal response can impair performance on a simple motor task. J App Physiol. 2001; 91: 821-831.
8. Bernstein DA, Carlson CR, Schmidt JE. Progressive relaxation: abbreviated methods. En: Lehrer PM, Woolfolk RL, Sime WE. Principles and practice of stress management. New York: The Guilford Press; 2007. 88-122.
9. Tarvainen MP, Niskanen JP, Lipponen JA, Ranta-aho PO, Karjalaien PA. Kubios HRV-Heart Rate Variability analysis software. Comput Methods Programs Biomed. 2014; 113 (1): 210- 220.
